# Vitamin K2 cannot substitute Coenzyme Q_10_ as electron carrier in the mitochondrial respiratory chain of mammalian cells

**DOI:** 10.1038/s41598-019-43014-y

**Published:** 2019-04-25

**Authors:** Cristina Cerqua, Alberto Casarin, Fabien Pierrel, Luis Vazquez Fonseca, Giampiero Viola, Leonardo Salviati, Eva Trevisson

**Affiliations:** 1 Clinical Genetics Unit, Department of Women’s and Children’s Health, University of Padova, Via Giustiniani 3, 35128 Padova, Italy; 2 Istituto di Ricerca Pediatrica IRP Città della Speranza, Corso Stati Uniti 4, 35127 Padova, Italy; 3 Univ. Grenoble Alpes, CNRS, CHU Grenoble Alpes, Grenoble INP, TIMC-IMAG, 38000 Grenoble, France; 4 Pediatric Hematooncology Laboratory, Department of Women’s and Children’s Health, University of Padova, Via Giustiniani 3, 35128 Padova, Italy

**Keywords:** Enzyme mechanisms, Molecular medicine

## Abstract

Coenzyme Q_10_ (CoQ_10_) deficiencies are a group of heterogeneous conditions that respond to ubiquinone administration if treated soon after the onset of symptoms. However, this treatment is only partially effective due to its poor bioavailability. We tested whether vitamin K2, which was reported to act as a mitochondrial electron carrier in *D*. *melanogaster*, could mimic ubiquinone function in human CoQ_10_ deficient cell lines, and in yeast carrying mutations in genes required for coenzyme Q_6_ (CoQ_6_) biosynthesis. We found that vitamin K2, despite entering into mitochondria, restored neither electron flow in the respiratory chain, nor ATP synthesis. Conversely, coenzyme Q_4_ (CoQ_4_), an analog of CoQ_10_ with a shorter isoprenoid side chain, could efficiently substitute its function. Given its better solubility, CoQ_4_ could represent an alternative to CoQ_10_ in patients with both primary and secondary CoQ_10_ deficiencies.

## Introduction

The mitochondrial respiratory chain (MRC) is composed of four multienzymatic complexes embedded in the mitochondrial inner membrane (MIM) of eukaryotes and of two electron carriers, cytochrome *c* (cyt *c*) and Coenzyme Q_10_ (CoQ_10_) or ubiquinone.

CoQ is a lipophilic molecule present in all cell membranes; its structure consists of a benzoquinone ring bound to a hydrophobic polyisoprenic tail of variable length, depending on the different species (ten units in humans, nine in mice, six in yeast)^1^. Beyond its fundamental role as electron carrier, CoQ has many other functions, including its involvement in pyrimidine biosynthesis, cell growth and differentiation, counteraction of apoptosis, mitophagy, functional modification of mitochondrial uncoupling proteins^2,3^ and regulation of sulfide metabolism^4,5^; moreover, in its reduced form it is the only lipid-soluble antioxidant synthesized endogenously^6^.

CoQ biosynthesis requires a series of proteins encoded by *COQ* genes and has been extensively studied in bacteria and yeast^7,8^. Currently, the complete metabolic biosynthetic pathway remains to be elucidated^9,10^ and in humans thirteen genes required for CoQ_10_ biosynthesis have been identified so far^11^.

Defects of ubiquinone biosynthesis cause CoQ_10_ deficiencies, a group of clinically and genetically heterogeneous conditions^12–14^. Mutations in ten *COQ* genes have been associated with primary CoQ_10_ deficiencies^11,15^. Depending on the localization and extent of the defect, the clinical symptoms vary greatly: the brain, the cerebellum, the muscle and/or the kidney may be involved, usually resulting in complex disease patterns^16^. Fibroblasts and muscle of affected individuals present a decrease of combined activities of Complex I + III (CI + III) and II + III (CII + III)^17^ (two CoQ_10_ – dependent reactions).

Patients with primary CoQ_10_ deficiency respond well to ubiquinone oral administration^18–20^, but this approach is not completely effective, in part also because of the poor bioavailability of CoQ_10_ related to its extreme hydrophobicity^21,22^. Consequently, CoQ_10_ supplementation requires high doses (up to 30–50 mg/kg/day)^14^ and in the last years several studies have investigated the efficacy of water-soluble formulations and of different ubiquinone analogs^23–27^. Ubiquinol-10, the reduced form of CoQ_10_, was found to have the same efficiency at a lower dosage than its oxidized form in a patient with CoQ_10_ deficiency, due to its better bioavailability^28^. It was also shown to be more effective in ameliorating the phenotype of a CoQ-deficient mouse model with mitochondrial encephalopathy^29^. However, experience in patients with primary forms is currently limited. The CoQ_10_ precursor 4-hydroxybenzoic acid (4-HB) and its analogs 2,4-dihydroxybenzoic acid (2,4-diHB), 3,4-diHB and vanillic acid exhibit beneficial effects on CoQ_10_ deficient cells *in vitro*, and 2,4-diHB rescued the mutant phenotypes of two different mice models of ubiquinone deficiency^15,30–32^.

Vitamin K2 (menaquinone-4, MK-4) is an isoprenoid quinone like CoQ_10_, containing a naphtoquinone ring and shorter isoprenoid side chain. Thus, vitamin K2 is less lipophilic than CoQ_10_ and it acts as electron carrier in some bacterial species^33^. A previous study suggested that vitamin K2, similarly to CoQ_10_, contributes to electron transport in the MRC of *D. melanogaster*^34^ and hence it could represent a promising drug to treat mitochondrial diseases, and specifically defects in ubiquinone biosynthesis.

In this work we tested whether vitamin K2 and Coenzyme Q_4_ (CoQ_4_, an analog of CoQ_10_ with a shorter isoprenoid side chain) could substitute CoQ function in human and yeast cells with CoQ deficiency.

## Results

### Vitamin K2 is incorporated into human mitochondria

**Figure 1:**
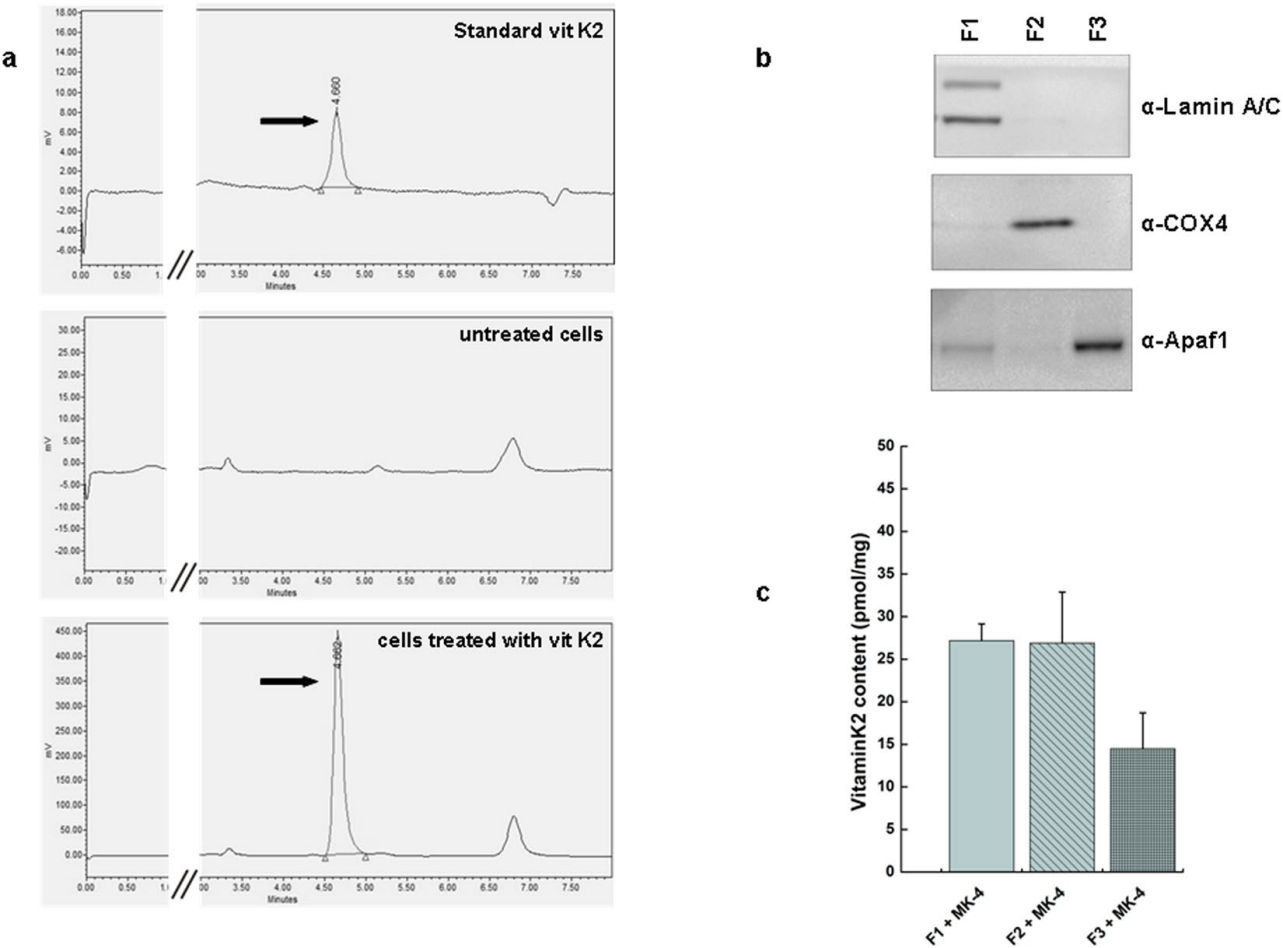
Vitamin K2 incorporates into human cells. (**a**) Chromatograms of a standard solution of vitamin K2 and its uptake in HeLa cells treated or not with MK-4 (5 μM) for 7 days. Cellular fractions of vitamin K2 were analyzed by reverse phase HPLC with ECD. The arrow indicates the peak corresponding to vitamin K2. (**b**) Proteins (50 μg) from subcellular fractions of HeLa treated with MK-4 (5 μM) for 7 days were separated by SDS–PAGE and immunoblotted with the indicated antibodies, specific for each fraction. F1 is constituted by nuclei and unbroken cells, F2 is an enriched mitochondrial fraction, and F3 includes cytosol, endoplasmic reticulum (ER), the other organelles and light membranes. (**c**) Mean ± s.e.m. (n = 3) of vitamin K2 content in cell lipid extracts prepared from each subcellular fraction was determined by HPLC with ECD.

In a first set of experiments, we checked whether vitamin K2 enters the cells and if it reaches mitochondria when it is added to the culture media as an aqueous solution. Cells were supplemented with vitamin K2 (5 μM) for 1 week and, after lipid extraction, vitamin K2 levels were determined by reverse phase HPLC with electrochemical detection (ECD). Untreated cells had no detectable vitamin K2, while in vitamin K2-treated cells the chromatogram displayed a clear peak at 4.7 min identical to the standard (Fig. 1a).

We then analyzed the subcellular distribution of vitamin K2. A cellular fractionation of MK-4-treated cells was carried out by differential centrifugation, resulting in three different fractions: F1 constituted by nuclei and unbroken cells, an enriched-mitochondrial fraction F2, and F3 that included cytosol, endoplasmic reticulum, Golgi apparatus, peroxisomes, lysosomes and the other light membranes. These fractions were analyzed by western blot using antibodies against Complex IV subunit 4 (COX4) as mitochondrial marker, lamin A/C as nuclear marker, and APAF1 as cytosolic marker (Fig. 1b). Lipid extracts were prepared from each fraction, and the incorporation of vitamin K2 was determined by HPLC. As shown in Fig. 1c, vitamin K2 accumulates in F1 and in mitochondria, confirming previous data^35^.

### Vitamin K2 does not restore electron flow in the respiratory chain of cells with CoQ10 deficiency

**Figure 2:**
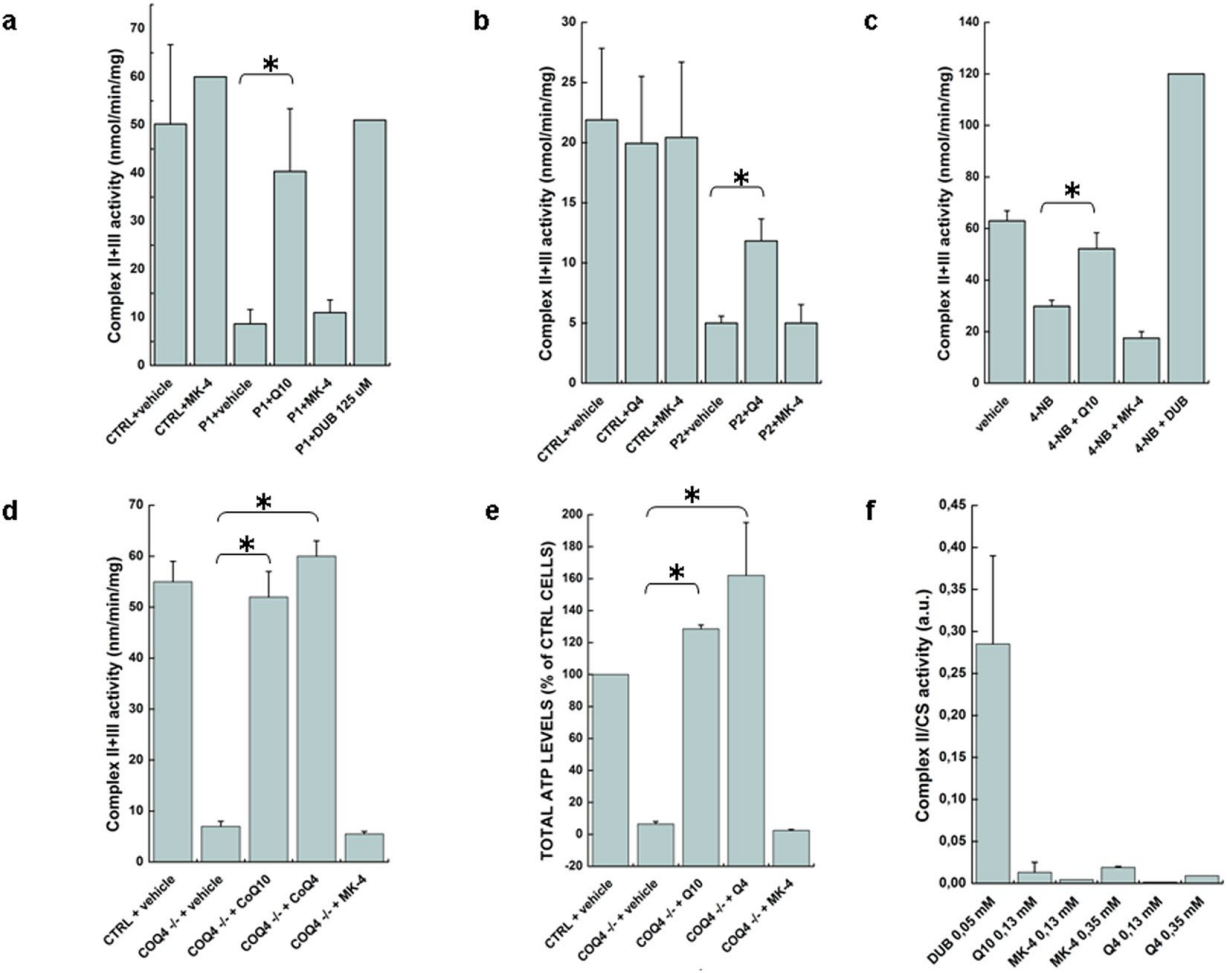
Vitamin K2 is not able to correct the defective MRC activities in human cells. (**a**) Complex II + III activity was determined spectrophotometrically in lysates obtained from CoQ_10_ deficient patient fibroblasts (P1) treated for 7 days with or without CoQ_10_ or MK-4 (5 μM), or with DUB (150 μM) added to the cuvette. Data are represented as mean ± s.e.m. (n = 3). (**b**) CoQ_10_ deficient patient fibroblasts (P2) treated for 7 days with or without CoQ_4_ or MK-4 (5 μM). Data are represented as mean ± s.e.m. (n = 3). (**c**) HEK293 cells treated with 4-NB (4 mM) with or without CoQ_10_ or MK-4 (5 μM) for 7 days (E). Data are represented as mean ± s.e.m. (n = 4). (**d**) HEK293 *COQ4*^−/−^ cells treated for 7 days with or without CoQ_10_, CoQ_4_ or MK-4 (5 μM). Data are represented as mean ± s.e.m. (n = 3). (**e**) ATP content was measured in HEK293 cells (CTRL) and in *COQ4*^−/−^ cells treated with 5 μM CoQ_10_, MK-4 or CoQ_4_ for 7 days. Data are represented as mean ± s.e.m. (n = 3). (**f**) Complex II activity was determined spectrophotometrically in lysates obtained from mitochondrial-enriched fractions of HEK293 cells by using DUB, CoQ_10_ or vitamin K2 as electron acceptors. Data are represented as mean ± s.e.m. (n = 3).

We evaluated the effects of vitamin K2 supplementation on CII + III combined activities in CoQ_10_ deficient fibroblasts with mutations in *COQ2* (P1)^36^. P1 fibroblasts were incubated with 5 μM CoQ_10_ or vitamin K2 for one week. CII + III activity was determined spectrophotometrically by following the reduction of 50 μM cyt *c* at 550 nm. As shown in Fig. 2a, untreated fibroblasts had 20% residual CII + III activity relative to controls (these cells harbor a hypomorphic allele that allows a residual production of CoQ_10_^37^). Only CoQ_10_-treated cells showed a normalization of mitochondrial CII + III activity, whereas vitamin K2 had no effect. As a further control we employed the more soluble decyl-ubiquinone (DUB) that could largely rescue the CII + III enzymatic defect when added to the cuvette.

To exclude a mutation-specific effect, we repeated the experiment using fibroblasts (P2) with a different genetic defect in *COQ2*^38^, and similar results were obtained. In this case we also tested the effect of CoQ_4_, a short chain quinone that is less lipophilic than CoQ_10_ (the isoprene chain is similar to that of vitamin K2). Supplementation with 5 μM CoQ_4_ for 1 week was able to restore normal CII + III activity (Fig. 2b).

We then employed a different system to induce CoQ_10_ deficiency. HEK293 cells were treated with 4 mM 4-nitrobenzoate (4-NB), a selective inhibitor of CoQ_10_ biosynthesis^39^, for 7 days. This treatment decreased CII + III activity by ~50%, and the addition of CoQ_10_ could rescue MRC functionality, whereas we did not observe any effect after vitamin K2 supplementation (Fig. 2c).

In the meantime, we were able to obtain a human cell line harboring a genetic ablation of *COQ4* (*COQ4*^−/−^)^40^. We therefore tested the effect of vitamin K2 also in this model (which is more stable than primary fibroblasts or 4-NB-treated cells). Cells were treated with 5 μM CoQ_10_, CoQ_4_ or vitamin K2 for 1 week and CII + III activity was determined as above. *COQ4*^−/−^ cells displayed a strong decrease of CII + III activity compared to controls (Fig. 2d). Only cells supplemented with CoQ_10_ or CoQ_4_ displayed a rescue of the biochemical defect.

Finally, we also analyzed the ATP content in *COQ4*^−/−^ cells treated with 5 μM CoQ_10_, vitamin K2 or CoQ_4_ for one week. (Fig. 2e). While CoQ_10_ and CoQ_4_ were largely able to rescue the bioenergetic defect, increasing ATP to levels comparable to control cells, vitamin K2 was not effective. To further support these results, we repeated the same experiment using patient’s fibroblasts with CoQ_10_ deficiency (P1) and obtained similar results (see Supplementary Fig. S1).

To test whether treatment with CoQ_4_ might affect CoQ_10_ content, we also measured CoQ_10_ levels in HEK293 cells treated for 7 days with 5 μM CoQ_10_, vitamin K2 or CoQ_4_. As expected, CoQ_10_ content was increased in cells treated with CoQ_10,_ while it was essentially unchanged in cells treated with either vitamin K2 or CoQ_4_, suggesting that the two compounds are not affecting CoQ_10_ biosynthesis (see Supplementary Fig. S2).

### Vitamin K2 does not restore respiratory growth of yeast *COQ* mutants

In a final set of experiments, we employed the *S. cerevisiae* Δ*COQ6* strain as additional model to test the effect of exogenous vitamin K2 on lower eukaryotes. This strain is unable to grow on non-fermentable carbon sources (ethanol and glycerol) if not supplemented with coenzyme Q_6_ (CoQ_6_)^41^. The Δ*COQ6* strain was transformed with the WT y*COQ6* as control or its mutant versions: the catalytically inactive but structurally stable F455X allele^42^ and the G386A_N388D double mutant^43^. Yeast growth was analyzed on a medium containing glycerol as a non-fermentable carbon source (YPG), with or without 50 μM vitamin K2. Vitamin K2 was not able to rescue the defective growth in the Δ*COQ6* strain (see Supplementary Fig. S3).

To exclude that the lack of rescue by exogenous vitamin K2 could be due to the inability of the compound to reach yeast MIM, we treated wild-type yeast with 50 μM vitamin K2 for 2 days and quantified vitamin K2 content by HPLC separation and ECD, after mitochondria purification and lipid extraction. As in human cells, we found that yeast mitochondria also uptake vitamin K2 significantly (see Supplementary Fig. S3).

Overall, these findings indicate that vitamin K2 is not active as electron carrier in the MRC of *S*. *cerevisiae*, or in that of human cells.

### Vitamin K2 is not an efficient substrate for complex II in human cells *in vitro*

Finally, we repeated the experiment performed by Vos and coworkers to test the electron acceptor capability of vitamin K2 compared to CoQ_10_. However, we also included as further controls DUB, a good electron acceptor for Complex II (CII) *in vitro*, and CoQ_4_. We measured CII activity by following the reduction of the 2,6-dichlorophenolindophenol (DCPIP) at 600 nm in mitochondrial-enriched fractions from HEK293 cells. As seen in Fig. 2f, neither CoQ_10_ nor vitamin K2 are good substrates for this reaction, since the activity measured with these compounds is almost two orders of magnitude inferior to that measured with DUB, at comparable concentrations.

## Discussion

Primary CoQ_10_ deficiency is one of the few treatable mitochondrial disorders, since oral supplementation with CoQ_10_ improves the symptoms of patients with different *COQ* genes defects^44^. Moreover, CoQ_10_ has been widely used for the treatment of patients with mitochondrial disorders because they frequently display secondary deficiency^45^. However, CoQ_10_ bioavailability and mitochondrial targeting are low due to its high hydrophobicity. Therefore, in the last years many efforts have been made to identify more water-soluble analogs of CoQ_10._ The use of certain oxidized or reduced CoQ_10_ dosages and formulations is able to increase ubiquinone levels in all human tissues after oral administration^46^. Also the use of 4-HB analogs is a promising strategy to bypass defective steps in the CoQ_10_ biosynthetic pathway^47^.

Vos and coauthors have proposed that vitamin K2 can function as an electron carrier in the MRC of a multicellular eukaryote, *D. melanogaster*^34^. The structure of vitamin K2 is similar to CoQ_10_, but it has a shorter hydrophobic carbon chain tail with four prenyl units that confer higher hydrophilicity. Because of the better bioavailability of vitamin K2, these findings opened the possibility that it could substitute CoQ_10_ in deficient patients.

To test this hypothesis, we have employed cells in which CoQ_10_ biosynthesis was disrupted or inhibited either by genetic or pharmacological means. Our results do not support the notion that vitamin K2 can act as an electron carrier in eukaryotic cells. In fact, even though it can easily reach mitochondria, vitamin K2 could not restore either electron flow or ATP biosynthesis in CoQ_10_-deficient cells.

One of the experiments performed by Vos and coworkers to support their claims showed that CoQ_10_ and vitamin K2 act as electron acceptors from complex II *in vitro*^34^. However, we now show that neither compound is a good *in vitro* substrate for Complex II compared to what is considered a good one, DUB^48^. In fact, the ability of vitamin K2 to accept electrons is about two orders of magnitude lower than that of DUB, and the situation for CoQ_10_ is only slightly better. Therefore, it is impossible to draw any conclusions from this experiment.

In agreement with our results, vitamin K2 failed to rescue mouse embryonic fibroblasts (MEFs) that are deficient for MCLK1 (the orthologue of human COQ7), where it could not restore the respiratory defect caused by CoQ_10_ deficiency^24^.

One of the limitations of our study is that we could not use *D. melanogaster* cells to exclude that the different results obtained in that model could be due to a species-specific effect. Nevertheless, our data clearly show that the role of vitamin K2 as an electron carrier (if confirmed) is probably restricted to Drosophila, and is not a general phenomenon in eukaryotic cells.

Finally, we found that CoQ_4_ supplementation rescues both CII + III activity and ATP content in CoQ_10_ deficient cells. It was previously shown that CoQ_2_ cannot mimic CoQ_10_ function in the MRC of human fibroblasts with ubiquinone deficiency^49^. Our data highlight that the effectiveness of electron transport in the MRC critically depends on the length of the isoprenoid chain, suggesting that two additional isoprenoid groups are sufficient to ensure the interaction with respiratory complexes binding sites. However, the mechanisms underlying these differences remain to be elucidated.

Although short chain quinones are toxic, this is particularly true for compounds with 0–3 isoprene units, whereas CoQ_4_ displays only minimal toxicity, evident only at concentrations of around 200 μM^50^, 40 times higher than those employed in this study.

Our findings indicate that CoQ_4_ could provide an interesting alternative to CoQ_10_ for the treatment of CoQ_10_ deficiency in humans. Further studies on animal models are warranted to unravel this issue.

## Materials and Methods

### Cell culture and chemicals

HEK293, HeLa cells and human fibroblasts were cultured in Dulbecco’s Modified Eagle Medium (DMEM, Invitrogen) supplemented with 10% fetal bovine serum (FBS, Invitrogen), 2 mM L-glutamine, 75 U/mL penicillin, 50 µg/mL streptomycin (Invitrogen) at 37 °C in a 5% CO_2_ incubator. Fibroblasts of patients with CoQ_10_ deficiency were previously described^38,51^. They had been obtained with the informed consent of the parents of the patients. The study was reviewed by the local ethical committee (Comitato Etico per la Sperimentazione Clinica della Provincia di Padova) – protocol AOPD2019 #0020044). All experiments were performed in accordance with the relevant guidelines and regulations of the University of Padova.

HEK293 *COQ4*^−/−^ were generated in our laboratory as described^40^ and were cultured in the presence of pyruvate (1 mM) and uridine (50 μg/mL) with 4% FBS, to minimize CoQ_10_ content in the medium.

For MRC activity and ATP measurement, cells were cultured for 2 days in DMEM medium containing low glucose (2 mM), to force mitochondrial respiration.

CoQ_10_, MK-4, CoQ_4_ and DUB were purchased from Sigma (Milan, Italy). They were dissolved in ethanol and added to the cells as less than 1% of total cell culture volume.

### Cell lysates, isolation of mitochondria, western blot, biochemical assays and ATP measurements

Whole-cell lysates were prepared in CHAPS buffer (1% CHAPS, 100 mM KCl, 50 mM HEPES pH 7.5, 1 mM EGTA) supplemented with the complete protease-inhibitor mixture (Sigma). Isolation of mitochondria was performed as detailed elsewhere^52^. Proteins were quantified using the Bradford method (Biorad) and fifty micrograms of proteins were separated by SDS-PAGE. Gels were probed with the following antibodies: COX4 (Thermo Fisher), Lamin A/C (Santa Cruz, USA), Apaf1 (Vinci-Biochem) and secondary HRP-conjugated anti-mouse, anti-rabbit or anti-goat antibodies (Santa Cruz, USA). Visualization of antibody protein complexes was achieved by enhanced chemiluminescence (LiteAblot Turbo, Euroclone) and the ChemiDocTM XRSþSystem (Bio-Rad). The enzymatic activity of RC complexes and citrate synthase (CS) was measured spectrophotometrically as previously described^53^, using a Cary UV 100 spectrophotometer (Varian). Where indicated, RC activities were normalized to CS. Cellular ATP was measured using the ATPlite kit (PerkinElmer), following the manufacturer’s instructions. ATP levels were normalized by total protein concentration.

### Lipid extraction and measurement of vitamin K2 by HPLC

A mixture of ethanol:hexane (2:5) was added to samples treated with MK-4 and after vortexing for 1 min, they were centrifuged at 2000 g for 5 min at 4 °C. The upper phase was recovered and dried. Lipid extracts were suspended in 150 μl of methanol prior to HPLC injection. Lipid components of the extracts were separated with a Waters Alliance e2695 HPLC system equipped with a vacuum degasser and an electrochemical detector (Antec Decade II). Chromatographic separations were conducted on a C18 4.6 × 150 mm analytical column, 3.5 µm particle size (Varian, Palo Alto, California) maintained at 30 °C. The pump flow rate was 1 mL/min and injection volume was comprised between 2 µL (human cells) and 10 µL (yeast cells). The separation was performed by isocratic elution with a mobile phase constituted by 96% methanol + 4% ethanol and 0.1 M LiClO_4_. The standard solution with vitamin K2 was injected to generate a standard curve that was used to quantify vitamin K2.

### Statistical analysis

Results are expressed as the mean ± SEM values of the indicated number (n) of independent experiments. Statistical significance was determined by unpaired Student’s t test between the indicated samples and differences were considered statistically significant for p < 0.05.

## Supplementary information

Supplementary File

## Data Availability

All data analyzed during this study are included in this published article and supplemental materials.
