# Supplementary material for: Vitamin K2 cannot substitute Coenzyme Q10 as electron carrier in the mitochondrial respiratory chain of mammalian cells
Source: Sci Rep. 2019 Apr 25;9:6553. doi: 10.1038/s41598-019-43014-y (PMC6484000; doi:10.1038/s41598-019-43014-y)
Supplement: Supplementary file 1 — Supplementary File [file 41598_2019_43014_MOESM1_ESM.pdf]

Vitamin K2 cannot substitute Coenzyme Q_{10} as electron carrier in the mitochondrial respiratory chain of mammalian cells

Cristina Cerqua[a,b], Alberto Casarin[a,b], Fabien Pierrel[c], Luis Vazquez Fonseca[a,b], Giampiero Viola[b,d], Leonardo Salviati[a,b*] and Eva Trevisson[a,b*]

[a] Clinical Genetics Unit, Department of Women's and Children's Health, University of Padova, Via Giustiniani 3, 35128 Padova, Italy

[b] Istituto di Ricerca Pediatrica IRP Città della Speranza, Corso Stati Uniti 4, 35127 Padova, Italy

[c] Univ. Grenoble Alpes, CNRS, CHU Grenoble Alpes, Grenoble INP, TIMC-IMAG, 38000, Grenoble, France

[d] Pediatric Hematooncology Laboratory, Department of Women's and Children's Health, University of Padova, Via Giustiniani 3, 35128 Padova, Italy,

[*]Send correspondence to:

Eva Trevisson, MD, PhD

Clinical Genetics Unit, Department of Women’s and Children's Health

University of Padova

Via Giustiniani 3, 35128 Padova, Italy

Phone: +39 049 8211402

Fax: +39 049 8211425

e-mail: eva.trevisson@unipd.it

Or

Leonardo Salviati

Clinical Genetics Unit, Department of Women’s and Children's Health

University of Padova

Via Giustiniani 3, 35128 Padova, Italy

Phone +390498217773

Fax: +39 049 8211425

E-mail: leonardo.salviati@unipd.it

Fig. S1

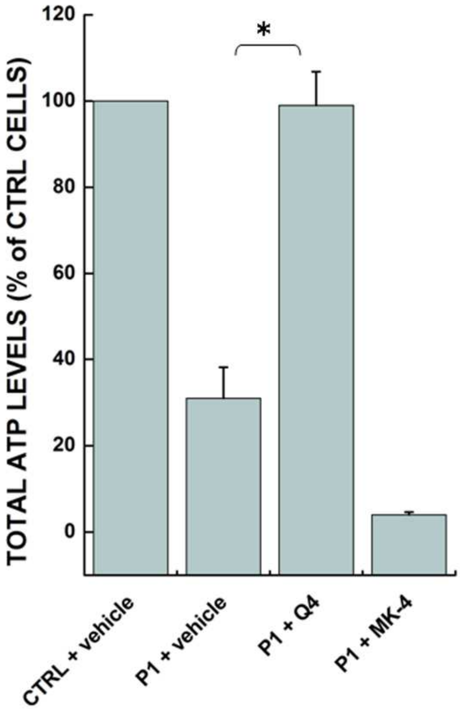

Figure S1. **CoQ_4 is able to rescue the decreased ATP levels of P1 fibroblasts, while vitamin K2 is not effective**.

ATP content was measured in control (CTRL) and P1 fibroblasts treated with 5 μM MK-4 or CoQ_4 for 7 days. Data are represented as mean ± s.e.m. (n=3).

Fig. S2

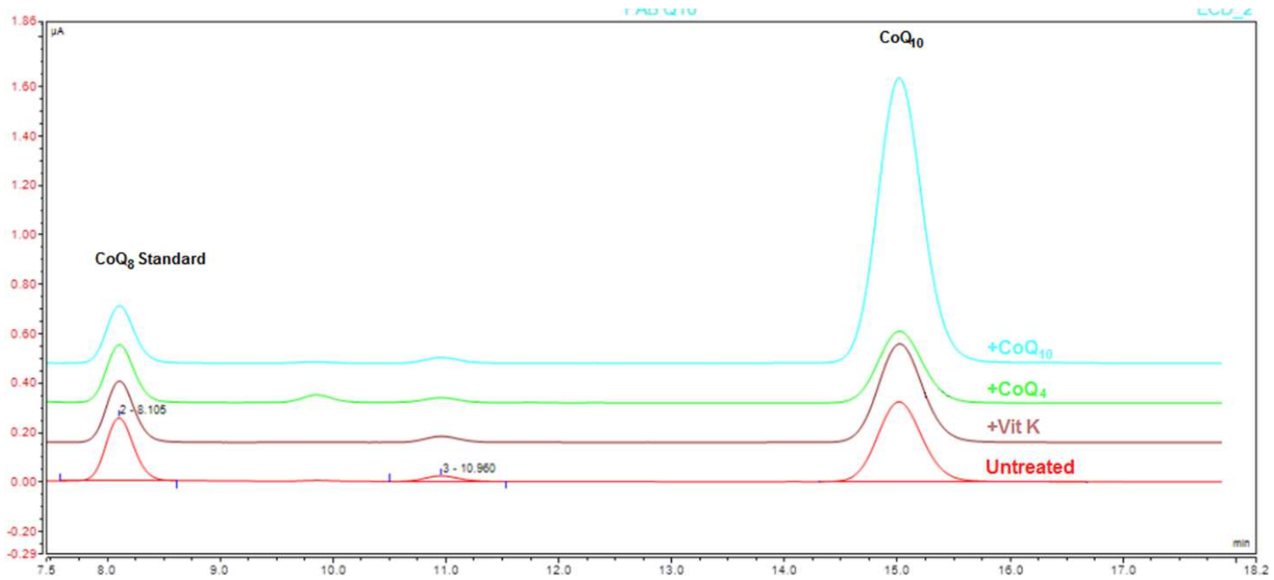

Fig. S2. **Supplementation with CoQ_4 or MK-4 does not affect the cellular levels of CoQ_{10}.** HPLC measurement of CoQ_{10} levels in lipid extracts from HEK293 cells treated for 7 days with or without CoQ_{10}, CoQ_4 or MK-4 (5 µM) (see text for details).

Fig. S3

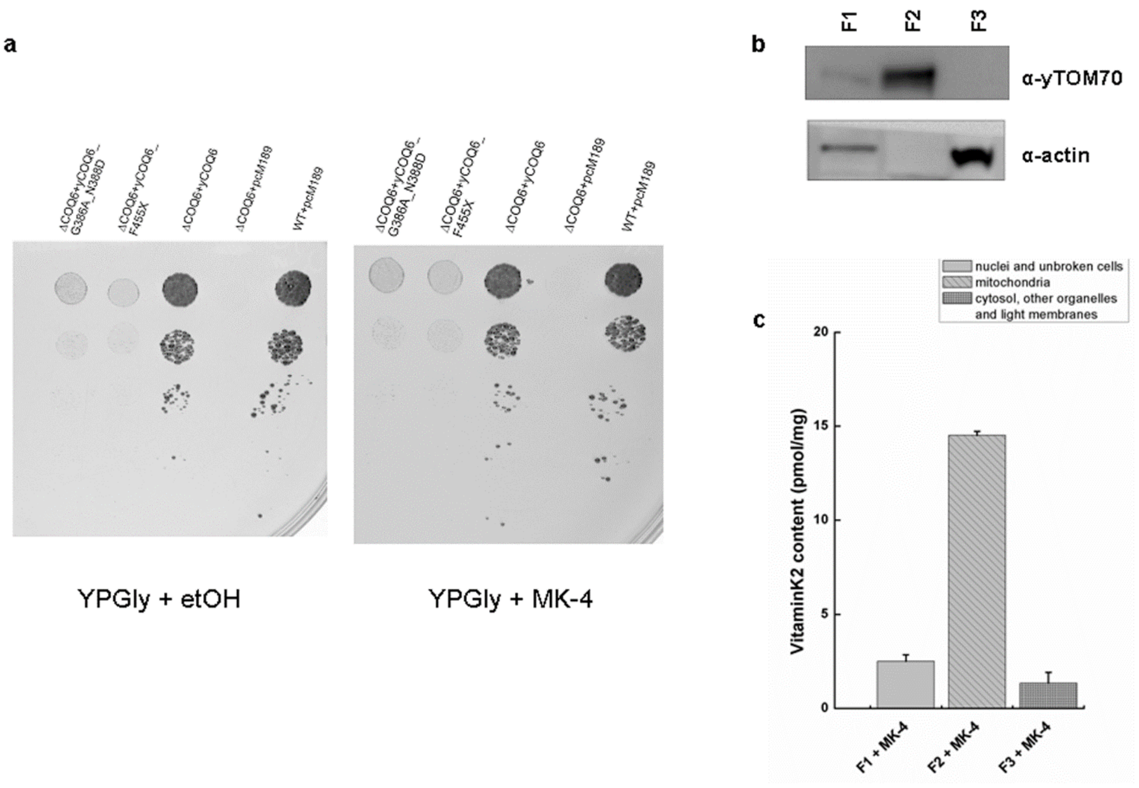

Figure S3. **Yeast *COQ6* mutants growth on a non-fermentable carbon source is not rescued by the addition of MK-4 to the medium**.

a) Wild-type and Δ*COQ6* yeast cells were transformed with the empty vector (pCM189) or the plasmids expressing the wild-type or two distinct mutant versions of y*COQ6* (G386A_N388D or F455X) and grown on YPG medium with or without 50 μM vitamin K2.

b) Proteins (50 μg) from fractions of wild-type yeast cells treated with MK-4 (50 μM) for 2 days were separated by SDS–PAGE and immunodecorated with antibodies against the mitochondrial protein yTOM70 (a kind gift from Dr. Kai Stefan Dimmer, University of Tubingen, Tubingen, Germany) and actin (Millipore), specific for the F3 fraction. F1 is constituted by nuclei and unbroken cells, F2 is an enriched mitochondrial fraction, and F3 includes cytosol, endoplasmic reticulum (ER), the other organelles and light membranes.

c) Mean ± s.e.m. (n=3) of vitamin K2 content in cell lipid extracts prepared from each fraction was determined by HPLC with ECD.

Methods

Lipid extraction and measurement of CoQ_{10} by HPLC

Harvested cells (5-6 mg protein) were resuspended in 0.3 mL of a 0.15 M KCl solution. Then, 200 uL glass beads, 10 uL of a 5 μM CoQ_8 standard solution and 3 mL methanol were added. The tubes were vortexed for 1 min, 2 mL petroleum ether (40-60° boiling range) were added and vortex was repeated for 1 min. The tubes were centrifuged at 700 rpm for 1 min, the upper phase was collected and the methanol phase was extracted again with 2mL petroleum ether. Both petroleum ether phases were combined, dried under a nitrogen flow and the lipid extracts were resuspended in 200 μL ethanol. HPLC analysis was conducted essentially as described [1] with the following modifications. 40 μL extracts were injected onto the C18 column and separation was obtained at a flow rate of 1 mL/min with a mobile phase composed of 50% methanol, 40% ethanol and 10% (90% (v/v) isopropanol, 10% (v/v) 1 M ammonium acetate, 0.1% (v/v) formic acid). The pre-column electrode (5020 guard cell, Thermo) was set at +650 mV and the post-column 5011A analytical cell at E1, -650 mV; E2, +650 mV. For each sample, the recovery was calculated based on the CoQ_8 standard, and CoQ_{10} was quantified based on a standard curve generated with commercial CoQ_{10}.

Yeast strains, media, transformations and isolation of mitochondria

Yeast strains WT BY4741 (*Mat a; his3Δ1; leu2Δ0; met15Δ0; ura3Δ0*) and Δ*COQ6* BY4741 (*Mat a; his3Δ1; leu2Δ0; met15Δ0; ura3Δ0; YGR255c::kanMX4)* were purchased from the Euroscarf Consortium (Frankfurt, Germany) and were cultured in rich YPD (1% yeast extract, 2% peptone and 2% glucose), YPG (1% yeast extract, 2% peptone and 3% glycerol) or selective SM GLU HLM – pABA medium (0.17% yeast nitrogen base without amino acids, 0.5% ammonium sulfate, 2% glucose or galactose) at 30°C. All yeast DNA transformations were performed with the PEG–

lithium acetate method as previously described [2]. The plasmids pcM189y*COQ6*_F455X and pRS416y*COQ6*_N386A_N388D were previously generated as reported in [3, 4]. For solid medium vitamin K2 was added to molten agar cooled to 50°C prior to pouring plates. Mitochondria from yeast cells were purified as previously described [2]. Vitamin K2 entrance in yeast cells was measured by HPLC, as explained in the section 4.3 of the main Article text.

References

1. Ozeir, M. et al. Coq6 is responsible for the C4-deamination reaction in coenzyme Q biosynthesis in Saccharomyces cerevisiae. *J Biol Chem* **290**, 24140-51.
2. Rowley, N. et al. Mdj1p, a novel chaperone of the DnaJ family, is involved in mitochondrial biogenesis and protein folding. *Cell* **77**, 249-59 (1994).
3. Ozeir, M. et al. Coenzyme Q biosynthesis: Coq6 is required for the C5-hydroxylation reaction and substrate analogs rescue Coq6 deficiency. *Chem Biol* **18**, 1134-42.
4. Heeringa, S.F. et al. COQ6 mutations in human patients produce nephrotic syndrome with sensorineural deafness. *J Clin Invest* **121**, 2013-24.
